# Impact of SARS-CoV-2 Infection on Cognitive Function: A Systematic Review

**DOI:** 10.3389/fpsyt.2020.621773

**Published:** 2021-02-10

**Authors:** Yazen Alnefeesi, Ashley Siegel, Leanna M. W. Lui, Kayla M. Teopiz, Roger C. M. Ho, Yena Lee, Flora Nasri, Hartej Gill, Kangguang Lin, Bing Cao, Joshua D. Rosenblat, Roger S. McIntyre

**Affiliations:** ^1^Mood Disorders Psychopharmacology Unit, University Health Network, Toronto, ON, Canada; ^2^Department of Psychological Medicine, Yong Loo Lin School of Medicine, National University of Singapore, Singapore, Singapore; ^3^Department of Affective Disorder, the Affiliated Brain Hospital of Guangzhou Medical University (Guangzhou Huiai Hospital), Guangzhou Medical University, Guangzhou, China; ^4^Laboratory of Emotion and Cognition, the Affiliated Brain Hospital of Guangzhou Medical University (Guangzhou Huiai Hospital), Guangzhou Medical University, Guangzhou, China; ^5^Key Laboratory of Cognition and Personality, Faculty of Psychology, Ministry of Education, Southwest University, Chongqing, China; ^6^Department of Psychiatry, University of Toronto, Toronto, ON, Canada; ^7^Department of Pharmacology, University of Toronto, Toronto, ON, Canada; ^8^Brain and Cognition Discovery Foundation, Toronto, ON, Canada

**Keywords:** neurotropism, cognitive function, delirium, depression, neuroinflammation, cytokines, COVID-19, brain health

## Abstract

The prevalence and etiology of COVID-19's impact on brain health and cognitive function is poorly characterized. With mounting reports of delirium, systemic inflammation, and evidence of neurotropism, a statement on cognitive impairment among COVID-19 cases is needed. A substantial literature has demonstrated that inflammation can severely disrupt brain function, suggesting an immune response, a cytokine storm, as a possible cause of neurocognitive impairments. In this light, the aim of the present study was to summarize the available knowledge of the impact of COVID-19 on cognition (i.e., herein, we broadly define cognition reflecting the reporting on this topic in the literature) during the acute and recovery phases of the disease, in hospitalized patients and outpatients with confirmed COVID-19 status. A systematic review of the literature identified six studies which document the prevalence of cognitive impairment, and one which quantifies deficits after recovery. Pooling the samples of the included studies (total sample *n* = 644) at three standards of quality produced conservative estimates of cognitive impairment ranging from 43.0 to 66.8% prevalence in hospitalized COVID-19 patients only, as no studies which report on outpatients met criteria for inclusion in the main synthesis. The most common impairment reported was delirium and frequent reports of elevated inflammatory markers suggest etiology. Other studies have demonstrated that the disease involves marked increases in IL-6, TNFα, and IL-1β; cytokines known to have a profound impact on working memory and attention. Impairment of these cognitive functions is a characteristic aspect of delirium, which suggests these cytokines as key mediators in the etiology of COVID-19 induced cognitive impairments. Researchers are encouraged to assay inflammatory markers to determine the potential role of inflammation in mediating the disturbance of cognitive function in individuals affected by COVID-19.

## Introduction

The coronavirus disease 2019 (COVID-19) is a respiratory condition caused by the RNA virus known as severe acute respiratory syndrome coronavirus 2 (SARS-CoV-2). The disease can result in several complex syndromes due to far reaching and variable effects on the human body. The virus binds the angiotensin-converting enzyme 2 (ACE2) receptor (1) which induces its internalization (2) and begins its replication cycle (3). In many viral infections, immune cells detect pathogenic RNAs and activate the inflammatory response, which triggers wide-ranging effects that contain the spread of the pathogen (4). However, SARS-CoV-2 can overcome this containment, which results in a positive feedback loop between viral propagation and the release of cytokines/chemokines (5); the molecular signals that regulate inflammation. This mutual amplification causes the disease's characteristic cytokine storm; a destabilizing increase in circulating inflammatory cytokines. The inflammation storm caused by SARS-CoV-2 is the main reason the disease has far reaching physiological effects.

The disease course of COVID-19 involves the elevation of key cytokines such as interleukin-6 (IL-6), tumor necrosis factor-α (TNFα) (3), and interleukin-1β (IL-1β), among others (5). Convergent evidence from laboratory, clinical, and epidemiological studies suggest that the foregoing key cytokines, among several others, are produced in greater quantities when the active hormonal form of Vitamin D3 is low (6). Indeed, these findings have shown that Vitamin D3 deficiency is common among COVID-19 patients, and it has been known for decades that the biosynthesis of TNFα and IL-1β are reduced by calcitriol in a dose dependent manner (7). Furthermore, some of these cytokines can cross the blood brain barrier and prompt their own release from microglia (8). This amplification of the inflammatory signal in the CNS can bias the excitation-inhibition ratio toward excitation (9). The foregoing excitation may explain the disproportionate number of seizures in COVID-19 cases as compared to the typical incidence of seizures observed in intensive care units (ICUs) (10). Due to substantial sequence homology with better characterized coronaviruses, some have speculated that the virus might be neurotropic like many of its predecessors (11). Angiotensin-converting enzyme 2 (ACE2) receptors are expressed in both the nasal cavity and the CNS. Consequently, researchers have proposed that the virus traverses the cribriform plate and infects the brain (10).

The foregoing observations have prompted a recent wave of publications characterizing the neurological and mental health ramifications of SARS-CoV-2 infection (10–15). Although this literature adequately characterized the variety of COVID-19 related neuropsychological conditions, the data on the cognitive effects of the disease are insufficient, and these data are often reported ambiguously. For instance, one of the most widely cited studies on the neurological manifestations of COVID-19, Mao et al. (12), conflated the prevalence of somnolence with that of delirium, by reporting them jointly as “impaired consciousness.” This kind of nebulosity regarding cognitive outcomes is evident throughout the current COVID-19 literature and results are often confounded by pre-existing cognitive impairment. Nevertheless, several lines of research indicate that even peripheral viral infections or inflammatory signaling may affect cognitive function (16–19). Accordingly, the recognition of SARS-CoV-2 neurotropism (1, 10, 11, 20) as well as significant immune system activation (3, 5, 8) provides the basis for hypothesizing that COVID-19 patients may be susceptible to multi-dimensional cognitive impairments across the domains of the Research Domain Criteria (RDoC) framework (18).

In light of the aforementioned shortcomings of the extant literature, this review aimed to summarize the available knowledge of the impact of COVID-19 on cognition (i.e., herein, we broadly define cognition reflecting the reporting on this topic in the literature) during the acute and recovery phases of the disease, in hospitalized patients and outpatients with confirmed COVID-19 status. The prevalence of cognitive impairments among hospitalized COVID-19 adult cases has been quantified, and the most prevalent types of cognitive conditions have been reported. No studies which report on outpatients met criteria for inclusion in the main synthesis of the present study. Non-primary sources and publications with conspicuous signs of selective reporting (e.g., selected cases of cognitive impairment) have been excluded from the main synthesis and are referenced, either directly or indirectly, only as sources of etiological insight.

## Materials and Methods

This review has been registered on PROSPERO (ID: CRD42020201232) prior to its commencement and was conducted in accordance with the recommendations of the PRISMA statement (21). Much of the relevant methodological details were described and updated on PROSPERO throughout the review process.

### Search Strategy

A systematic search of the literature was conducted on CINAHL Plus, MEDLINE, EMBASE, and APA PsycINFO. A manual citation search was conducted in the reference lists of articles included in full-text screening. As shown in Table 1, the searches involved both the “cognition” and the “COVID-19” concepts on all databases. Functional synonyms were used for COVID-19, and the word “cognition” was truncated to include all variations of the term. Time of publication was restricted to the interval between 2019 and 26/08/2020. EMBASE search yielded numerous generic and irrelevant documents. To exclude these results, the EMBASE search was restricted to papers with the two concepts appearing within four words of each other. MEDLINE search yielded numerous generic results that did not report patient data. To exclude these results, the MEDLINE search was restricted to papers with the “COVID-19” and “patient” concepts appearing within four words of each other. All searches on all databases were only applied to the title, abstract, and related keyword fields. The OVID platform was used to search all databases, with the exception of CINAHL Plus, for which the EBSCOhost platform was used. Database-specific restrictions and keywords are shown in Table 1.

**Table 1:** Databases and associated search queries used in all systematic searches.

CINAHL Plus	(TI cogni^*^ OR AB cogni^*^) AND (TI COVID-19 OR TI COVID19 OR TI Sars-CoV-2 OR TI 2019 novel coronavirus OR TI coronavirus disease 2019)
MEDLINE	(cogni^*^.tw,kf.) AND (yr=“2019 -Current”) AND ((COVID-19 or COVID19 or Sars-CoV-2 or 2019 novel coronavirus or coronavirus disease 2019) adj4 (patient^*^ or individual^*^ or adult^*^ or person^*^ or man or woman or men or women))
EMBASE	((cogni^*^ adj4 (COVID-19 or COVID19 or Sars-CoV-2 or 2019 novel coronavirus or coronavirus disease 2019)).ti,ab.) AND (human and english language and yr=“2019 -Current”)
PsycINFO	(exp Executive Function/ or exp Cognition/ or exp Cognitive Impairment/ or cogni^*^.mp. or exp Social Cognition/) AND (yr=“2019 -Current”) AND (COVID-19 or COVID19 or Sars-CoV-2 or 2019 novel coronavirus or coronavirus disease 2019)

### Inclusion and Exclusion Criteria

To be included, studies were required to report either primary or secondary cognitive outcomes of SARS-CoV-2 infections confirmed by the presence of biological markers, as indicated by Reverse Transcriptase Polymerase Chain Reaction (RT-PCR) or antibody assays, of blood, cerebrospinal fluid (CSF), or oronasopharyngeal swabs. Studies that only reported on suspected COVID-19 cases or on patients under the age of 18 were excluded, along with publications that did not report explicitly on cognitive function as characterized by reliable medical tests (e.g., CAM) or DSM-IV/V criteria. Papers in languages other than English, and papers which reported the cognitive outcomes of the socioeconomic or cultural circumstances of the COVID-19 pandemic, were also excluded. Within the included samples, data from those with cognitive impairments known or suspected to have existed prior to infection, were omitted from data analysis wherever possible. Peer-reviewed letters, case series, case-control studies, retrospective chart reviews, cohort studies, and point prevalence studies were included for analysis. Reviews, perspective/position papers, protocols/study designs, editorials, individual cases, or any non-primary sources were excluded to minimize the risks of redundant data collection and publication bias.

In compliance with the PRISMA statement (21), this review has been conducted in accordance with the PI(E)COS outline below:

- **Participants:** Patients aged ≥18 years with no known pre-exisiting cognitive impairments.
- **Intervention:** No intervention was evaluated in the present review.
- **Exposure:** SARS-CoV-2 infection confirmed by the presence of biological markers, as indicated by Reverse Transcriptase Polymerase Chain Reaction (RT-PCR) or antibody assays, of blood, cerebrospinal fluid (CSF), or oronasopharyngeal swabs.
- **Comparator:** No overarching comparator applied to the present study, as assessments of cognitive function were categorical.
- **Outcome:** Prevalence of cognitive impairment during acute and recovery phases of COVID-19, as identified by the Confusion Assessment Method (CAM), 4 A's Test (4AT), DSM-IV/V criteria, or clinical diagnosis.
- **Studies:** Peer-reviewed case series, case-control studies, retrospective chart reviews, cohort studies, and point prevalence studies, which do not restrict selection to cognitively impaired patients.

### Data Extraction Protocol

In compliance with the PRISMA statement (21), articles were assessed for relevance by title and abstract screening conducted by three independent reviewers. Full texts were examined for relevance when titles and abstracts were uninformative. Deduplication, screening, and quality assessments were conducted on the Covidence platform for systematic review management (https://www.covidence.org/). Conflicts in judgement were either resolved by discussion or by the judgement of the third reviewer. Throughout the review process, publications were only advanced to the next phase of examination upon the agreement of at least two reviewers. One reviewer extracted the data, and the results of these extractions were closely inspected by the co-authors.

The extracted data included: first author, year of publication, study design, sample size, sex ratio, average age, location, diagnostic test or criteria, and the prevalence of cognitive impairments. The percent prevalence of impairments and mean age of the total sample were calculated as weighted averages of the corresponding values (i.e., percent prevalence values and average age of the constituent samples, with sample sizes as weights). “Impairment” was used as a broad umbrella term that included the following conditions: altered mental status (AMS), confusion, delirium, encephalitis, encephalopathy, psychosis, dysexecutive syndrome, or any other condition explicitly reported as entailing cognitive deficits.

### Methodological Quality Assessment

The quality assessment tool for case studies proposed by Murad et al. (22) was adapted to the final collection of articles of the present study. The adapted form used in the present study is presented in Table 2. The original tool assesses risks of bias with eight items across four domains: selection, ascertainment, causality, and reporting. Three items in the causality domain were omitted due to irrelevance; namely, the items for dose-response, challenge-rechallenge, and adequacy of time period from exposure/treatment till follow-up. Each included study was assessed by two reviewers, and conflicts were resolved by discussion. For domains in which judgments were necessarily made for separate participant subgroups, the weight of the associated domain was divided by the number of subgroups, and the sum of weights associated with items demonstrating low risk of bias was divided by the total number of items for a final quality score. Studies with scores ≤ 0.6 were considered to be at risk of being biased, and studies were ranked in accordance with this standard of quality.

**Table 2:** Risk of bias tool used in the present study.

Selection	1. Do the patients represent the whole experience of the investigator (center) or is the selection method unclear to the extent that other patients with similar presentation may not have been reported?
Ascertainment	2. Was the exposure adequately ascertained? 3. Was the outcome adequately ascertained
Causality	4. Were other alternative causes that may explain the observation ruled out?
Reporting	5. Is the case(s) described with sufficient details to allow other investigators to replicate the research or to allow practitioners to make inferences related to their own practice?

## Results

Seven studies which report on the prevalence of cognitive impairments associated with SARS-CoV-2 infection were included in this systematic review. The overall prevalence estimates from pooled and nested samples ranged from 43.0 to 66.8%, and one study demonstrated a correlation (*r* = 0.557, *p* = 0.002) between C-reactive protein (CRP) and reaction time in recovered COVID-19 patients (23). It is noteworthy that delirium was the most represented type of cognitive impairment in the prevalence estimates included. These conservative estimates along with the main findings of their associated studies, are summarized in **Table 4**.

### Systematic Search Results

**Figure 1:**
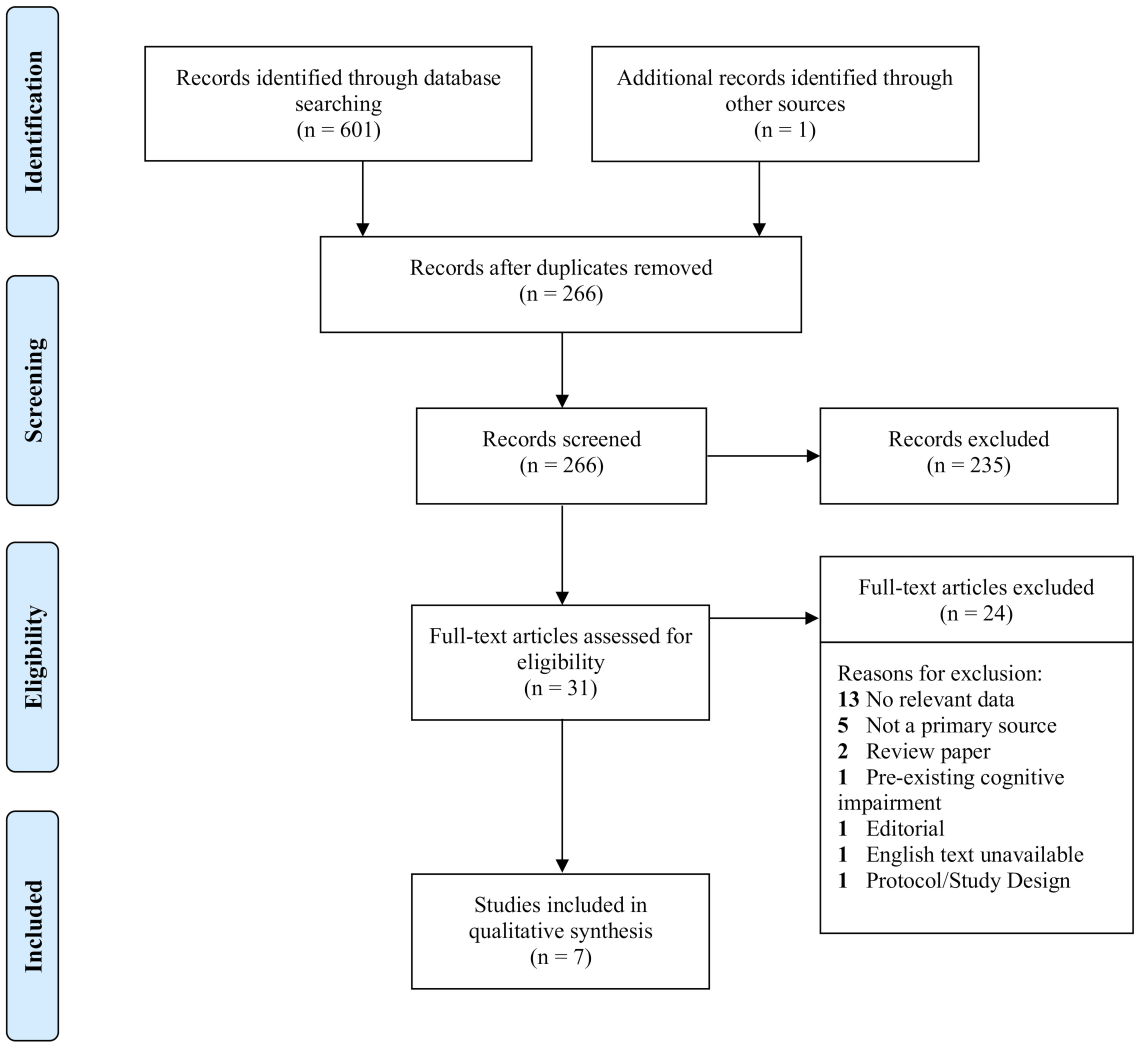
Flow diagram for the systematic review as per PRISMA criteria.

Due to the continuing publication of studies on COVID-19 and the scarcity of studies on cognition, databases were systematically searched at three time points: 19/07/2020, 09/08/2020, and 26/08/2020. Of 601 studies found in databases, 336 were identified as duplicates. After title and abstract screening of 266 studies, a total of 31 studies met criteria for full text assessment, which included one study found in a reference list. Of the 31, only seven met criteria for inclusion, and 24 studies were excluded for reasons listed in Figure 1. Six of the included studies reported the prevalence of cognitive impairment in COVID-19 patients during hospitalization, and one study (23) reported on cognitive function after recovery. The foregoing study was omitted from the total sample (*n* = 644) because the cases may not have been confirmed, and the impairments reported therein were not comparable to those in the other six studies. Nevertheless, the paper was included for its relevant findings, and because such few studies met requirements for inclusion. Notwithstanding, there was a significant lack of studies investigating the cognitive effects of SARS-CoV-2 infection. An informal search of the literature on October 11th, 2020 demonstrated that newer publications which discuss the COVID-19-cognition relationship mostly relied on the same studies found in the three formal searches of this review. Notably, Mao et al. (12) was cited often when relating infection to cognitive outcomes, but its methodological limitations necessitated its exclusion.

### Quality Assessment Results

Five of the included studies had satisfactory scores above the 0.6 threshold, and two were considered unsatisfactory. The quality assessment naturally resulted in three tiers of quality: two studies had scores above 0.8, three studies had identical scores of 0.8, and two studies had identical scores of 0.6. Table 3 lists the three tiers in order of decreasing quality, along with the domains in which each study was deemed to be methodologically lacking.

**Table 3:** Three-tiered ranking of included studies by quality assessment score.

**Study**	**Helms et al. (24) (B)**	**Pinna et al. (25)**	**Knopp et al. (26)**	**Varatharaj et al. (27)**	**Zhou et al. (23)**	**Mcloughlin et al. (28)**	**Helms et al. (29) (A)**
Quality score	1.0	0.9	0.8	0.8	0.8	0.6	0.6
High risk domain	n/a	Causality	Causality	Selection	Selection	Selection, causality	Reporting, causality

### Prevalence of Neurocognitive Impairments

All data gathered from the included articles are presented in Table 4, which also includes summaries of study methods, major findings, and limitations. The total sample was *n* = 644 and the weighted mean of the reported average ages from its constituent samples was 69 years (SD = 7.90). Of this sample, at least 43.0% (SD = 16.2) exhibited one of the following neurocognitive impairments: delirium, confusion, AMS, encephalopathy, encephalitis, or psychosis. However, this percentage involves one study (26) which included 72 patients with premorbid dementia. Nevertheless, this study produced relevant results from models corrected for dementia in a separate analysis (reported in Table 4). Upon omission of this study's sample (*n* = 217), at least 49.9% (SD = 15.8) of the remaining pooled sample (*n* = 427) were cognitively impaired (the same neurocognitive impairments reported in the overall sample), and the weighted mean age for this sample was 64 years (SD = 4.50). Upon exclusion of Zhou et al. (23) and Knopp et al. (26), the percent prevalence for the combined samples of the top two tiers of quality (*n* = 304) is 53.0% (SD = 20.6), and the weighted mean age was 65 years (SD = 6.01). The types of impairment remained the same after these exclusions, with the exception of confusion; however, the more severe form of this impairment (i.e., delirium) retained its status as the most represented. The percent prevalence for the sample of the top tier alone (*n* = 190) is 66.8% (SD = 6.57), with weighted mean age of 61 years (SD = 1.70); only delirium and AMS were reported in this sample. There are additional cases of cognitive impairment that have not been incorporated into these percentages. The prevalence of some such cases along with the types of impairments as well as reports on inflammatory markers are mentioned under “Descriptions” in Table 4. Overall, four studies reported on inflammatory markers [C-reactive protein (CRP) or IL-6] and all four publications reported elevations in at least one of these inflammatory markers which were concomitant with cognitive impairment. Helms et al. (24) and Knopp et al. (26) reported respective elevations of IL-6 and CRP in delirious patients. Pinna et al. (25) found elevations of CRP in cases of AMS, and Zhou et al. (23) found a positive correlation between reaction time and serum (CRP) (*r* = 0.557, *p* = 0.002).

**Table 4:** Data summaries of included publications.

**Study**	**Design**	**Sample *N*** **(confirmed**^***a***^**)**	**Female *n* (%)**	**Age Median or** $\bar{\mathrm{Mean}}$ **(range, IQR)**	**Location**	**Test**	**Description**^***b***^	**% Impaired**^***c***^
Pinna et al. (25)	Retrospective chart review	50 (50)	21 (42)	$\bar{59.6}$	Chicago, Illinois, USA	–	All cases were confirmed by RT-PCR of nasopharyngeal swabs, and the 50 cases were selected based on the availability of data on neurological status. The most common neurological feature of COVID-19 was AMS, affecting 30 patients in the sample. Twenty-four percent had cognitive abnormalities (mostly short-term memory loss), 40% had cerebrovascular issues (e.g., ischemic stroke, brain hemorrhages, transient ischemic attacks, etc.), and 26% had seizures. All reported measurements of C-reactive protein (CRP) were well above 70 mg/L. The main limitation of this study is that it does not present the extent to which these manifestations overlap in the sample, which makes it impossible to determine the number of patients with cognitive manifestations overall. It also omits all detail on the cognitive testing methods used, which makes it impossible to ascertain the effective definitions of these manifestations in context. Additionally, about half the sample exhibited neurological manifestations at least 24 h after admission, and the drugs given to this group are not listed.	60.0
Helms et al. (29) (A)	Case Series	58 (58)	–	63	Strasbourg, France	CAM, RASS	This case series includes ICU cases, all of which have been hospitalized for ARDS due to COVID-19 (confirmed by RT-PCR of nasopharyngeal swabs). Forty patients received a CAM-ICU test and 26 of them were positive for confusion/delirium. Sixty-nine percent of the total sample exhibited agitation as per the RASS, and of 39 patients tested for dysexecutive syndrome, 36% were positive. Unfortunately, only 14% of the sample were tested for neurological manifestations prior to treatment, and the paper does not specify the number of patients that exhibited cognitive symptoms in this subgroup. The sedatives used were propofol, midazolam, and sufentanil, all of which may affect cognition. Hence, the main limitation of this study is that the cognitive effects reported are confounded by treatment.	44.8
Helms et al. (24) (B)	Cohort Study	140 (140)	–	62	Strasbourg, France	CAM, RASS	The same researchers conducted a larger study to evaluate the prevalence and type of delirium seen in COVID-19 patients (confirmed as in the smaller study) in the ICU. One hundred and twenty-two of the patients were assessed for delirium with the CAM-ICU, whereas 14 died without being assessed (too sedated to respond), and four could not speak French. Of these 122 patients, 97 were positive for delirium which gives a 79.5% prevalence in ICU cases. However, a selection bias may be in effect because the unresponsive patients may have been cognitively intact prior to their deaths, so the number to the right reports a more conservative percent prevalence that includes the 18 unassessed patients. Furthermore, the authors found that 86.6% of delirious patients were hyperactive/agitated (RASS +3/+4). CSF analysis revealed inflammation in 64.3% of the assayed patients, one marker being IL-6. Twenty-two patients presented with either “delirium and/or corticospinal tract signs” at admission.	69.3
Varatharaj et al. (27)	Point Prevalence Study	153 (114)	44 (29)	71 (23–79)	United Kingdom	–	This study includes 153 COVID-19 cases, 114 of which were confirmed by PCR of nasopharyngeal swabs or CSF, or by antibodies in blood. The data to the left loosely applies to the confirmed case sample, as age and sex data were absent in many cases. Of the 114 confirmed cases, 16.6% were diagnosed with a psychiatric disorder (most of which were newly diagnosed), 12.3% were either diagnosed with psychosis or a dementia-like neurocognitive impairment, and 13.2% had either encephalitis or unspecified encephalopathy. Although most psychiatric disorders seemed to occur post-infection, they may have been undiagnosed but present prior to infection. This study does not distinguish iatrogenic effects from COVID-19 effects, and the treatments used were not described. Furthermore, a significant risk of confirmation bias is in effect because the data collection protocol was a deliberate search for neurological features of COVID-19.	29.8
Zhou et al. (23)	Case-Control Study	29 (0)	11 (37.9)	$\bar{47.00}$ (30–64)	Zhejiang, China	TMT, SCT, CPT, DST	This study tested several cognitive domains in recovered COVID-19 patients vs. controls using cognitive tests^*^ with good test-retest reliability in the Chinese population. Test scores have been shown to be affected by age, sex, ethnicity, and education level, so patient scores were compared to controls which were matched by these variables. Accordingly, criteria for all participants omitted any current or past psychiatric disorders, non-Han ethnicity, or having had <9 years of formal education. Furthermore, patients had to have at least two negative PCR results. Inflammatory markers were recorded to search for correlations with test scores. Recovered COVID-19 patients exhibited statistically significant (*P* < 0.05) reductions in items testing sustained attention, and (CRP) was correlated with CPT1 reaction time (*r* = 0.557, *p* = 0.002). The paper makes no mention of whether the patients were confirmed COVID-19 cases prior to supposed recovery, and the sample is unrepresentatively small. ^*^[Trail Making Test (TMT), Sign Coding Test (SCT), Continuous Performance Test (CPT), Digital Span Test (DST)]	–
Mcloughlin et al. (28)	Point Prevalence Study	71 (71)	20 (28.2)	$\bar{61}$ (24–91)	London, UK	4AT, DSM-IV	This study noted all-cause mortality, delirium, and the capacity to function in normal daily life in RT-PCR confirmed COVID-19 patients. All alert/responsive patients were assessed for delirium using DSM-IV criteria, the 4AT delirium screen, and medical notes from the past 24 h. Of the 71 patients, 24 were too sedated to give meaningful responses to the 4AT. The remaining 47 were effectively assessed for delirium, and six of them had dementia. Forty-two percent of the 47 had delirium, but 63.4% (*n* = 26) of this sample had delirium when those with dementia are excluded. However, the unresponsive patients may have been cognitively intact when they were not sedated, so the number to the right reports a more conservative percent prevalence that includes these 24 patients. At 4 week follow-up, there was no significant cognitive score^*^ difference between those who had delirium and those who did not. However, delirium was associated with poor daily functionality, which was measured by a composite score from both the NEADLS^*^ and the Barthel Index. Finally, delirium did not predict all-cause mortality when adjusted for age, sex, and frailty. Of note, the sample was too small, and the patients involved were at varying stages of the disease progression. ^*^TICS-m, modified Telephone Interview for Cognitive Status; NEADL, Nottingham Extended Activities of Daily Living Scale	40.0
Knopp et al. (26)	Prospective Cohort Study	217 (unknown)	83 (38)	80 (70–99, 74–85)	London, UK	–	In this study, the same group as that of the above study, aimed to quantify the same outcomes on a larger scale. In this iteration, only patients aged ≥70 years were included, and patients diagnosed with COVID-19 by a specialist infectious diseases team were included based on laboratory, radiological, and clinical findings, even if results for RT-PCR of oronasopharyngeal swabs were negative. Thirty-three percent (*n* = 72) of the sample had pre-existing dementia, and 29% of the sample had delirium suspected to be caused by COVID-19. The degree to which these two subgroups overlap has not been made clear, but in models adjusted for dementia, age, and other factors, delirium was associated with cognitive impairment at discharge (OR 44, 95% CI 7.4–260). Median CRP was 92 mg/L and	29.0
							CRP was negatively correlated with frailty. Furthermore, delirium was associated with mortality in this cohort (*p* < 0.001), unlike Mcloughlin et al. (28). Similar limitations apply to these two studies, and the limitation the group seems to emphasize most is that these data have all been collected in the same hospital, allowing very little generalizability due to homogeneity of conditions.	

a *Number of participants with confirmed COVID-19 status as per the confirmation standards outlined in the associated description*.

b *All studies report heterogenous data from hospitalized patients at different stages of acute-phase COVID-19; the sole exception being Zhou et al. (23), which reports on recovered cases*.

c *Includes participants exhibiting altered mental status (AMS), confusion, delirium, encephalitis, encephalopathy, or psychosis,; excludes patients with known pre-existing conditions that are principally characterized by cognitive impairments (e.g., dementia, mild cognitive impairment, traumatic brain injury, schizophrenia). For studies in which the overlap between these conditions was unknown, the available value which represents the most overt form of cognitive impairment was reported (i.e., delirium/confusion)*.

## Discussion

### Quality of Information

One of the limitations with respect to the interpretation of the available studies was that the medications prescribed to treat COVID-19 may have significantly confounded results. As suggested in Table 3, four of the included studies did not exclude confounds, the most significant of which was the dyscognitive effect of medications which may have been used to treat COVID-19 (e.g., steroids). Furthermore, much of the literature does not adequately separate cases with pre-existing neurocognitive impairments from cases of cognitive impairment associated with COVID-19. Stringent as the inclusion criteria were, these problems still presented themselves in the included studies to varying degrees. For instance, Knopp et al. (26) did not clarify whether some of the 72 participants with pre-existing dementia were included in the delirious subgroup. Dementia and delirium are often confused and misdiagnosed in clinical practice (30), and some evidence has suggested that patients with dementia are especially at risk of developing persistent delirium (31). This suggests that there may have been an overestimation of COVID-19 related delirium due to the inclusion of patients with dementia. There was also ambiguity in Knopp et al. (26) regarding the methods used to confirm SARS-CoV-2 infection. The article reports that these assessments were conducted by infectious disease experts but does not mention the exact methods used, or whether they were contested in the scientific literature. Nevertheless, this article was an exemplar of the fundamental challenges involved in gathering large datasets from the busy hospital environment. It is a testament to the difficulty of the situation that Knopp et al. (26) was one of the best studies available. The other included studies did allow for the exclusion of patients with known pre-existing cognitive impairments but had other significant limitations, as indicated in Tables 3, 4. It is likely due to such challenges that most studies did not quantify the extent of overlap between subgroups with different types of COVID-19-related cognitive impairments. In those cases, conservative prevalence statistics were produced, involving only the most severe and overt cognitive conditions (i.e., delirium).

### Implicit Reporting Bias in Prevalence Results

As mentioned in “Prevalence of Neurocognitive Impairments,” the prevalence statistics produced for various combinations of the included samples ranged from 43.0 to 66.8%. Although these numbers were calculated conservatively on the study level, a reporting bias may have been amplified by pooling the results. One of the limitations of extant literature is the non-publication of negative study results. In an analysis of 64 randomly selected scientific articles, out of 145 empirically supported potential determinants of selective reporting, it was found that the leading determinant was a “focus on preferred findings,” accounting for 36% of cases (32). Despite best efforts, this review may have implicitly amplified this type of bias. It is certainly possible that some of the excluded attempts to characterize the presentations of COVID-19 involved cognitive assessments that produced negative results. Aside from bias toward preferred findings, these results may not have been reported simply for the sake of brevity. Many of the foregoing studies considered throughout this review were very broad in scope, attempting to provide a complete impression of the COVID-19 syndrome. In such cases, the omission of negative results on cognitive assessments may have seemed prudent. This implicit risk of selective reporting is difficult to rectify and is a fundamental problem in the systematic review methodology. Furthermore, the unspecified diagnostic criteria in three of the included studies may have masked loose definitions of cognitive impairment, which may have resulted in the overestimation of the associated prevalence statistics. Taking these considerations in isolation, the 43.0–66.8% prevalence range may be viewed as non-representative of the real-world prevalence of COVID-19 induced cognitive impairments. However, considering the parsimonious neurobiological models which predict these impairments, the results included herein cannot be dismissed.

### Neurobiological Model for COVID-19 Related Cognitive Impairments

As mentioned in the introduction, COVID-19 involves elevations in IL-6, TNFα (3), and IL-1β (5), which are often exacerbated by Vitamin D3 deficiency (6). Furthermore, IL-6 and TNFα can cross the blood brain barrier and activate microglia (8). These activated cells release IL-1β, the receptors for which are especially concentrated in the postsynaptic compartments of hippocampal neurons (33). This renders the hippocampus especially vulnerable to IL-1β, which has been shown to disrupt long term potentiation (LTP) and memory (34). Other work has also suggested that attentional processes are subserved by hippocampal activity, demonstrating the importance of working memory in determining how attention is directed and sustained (35). Attention and working memory are among the principle cognitive functions impaired in delirium (30), and clinically manifested neurotropism may exacerbate this through additional pathways.

ACE2 acts as the functional and host receptor for coronaviruses (1) and regulates normal brain function by stimulating brain-derived neurotrophic factor (BDNF) activity (36). BDNF plays a critical role in attenuating microglial activation (37) and neuronal inflammation (38), and low BDNF levels are associated with cognitive impairment in both human and animal studies (37, 39, 40). SARS-CoV-2 is now known to decrease ACE2-mediated BDNF activity (20), possibly by acting as a competitive angiotensin-II-antagonist via spike protein-ACE2 binding. Regardless of the mechanism by which SARS-CoV-2 inhibits ACE2, the resulting reduction of BDNF is likely to cause cognitive impairment (20). Furthermore, the permeability of the blood-brain barrier (BBB) can be increased by IL-6 (41), which can further microglial activation by enhancing the CNS effects of serum cytokines. Astrocytic activation also contributes to the inflammatory signal in the CNS, which is especially pronounced when BBB integrity is compromised (41). Indeed, increased BBB permeability has been observed in COVID-19 patients (26), and high CRP/IL-6 concentrations are reported by several studies (24–26).

**Figure 2:**
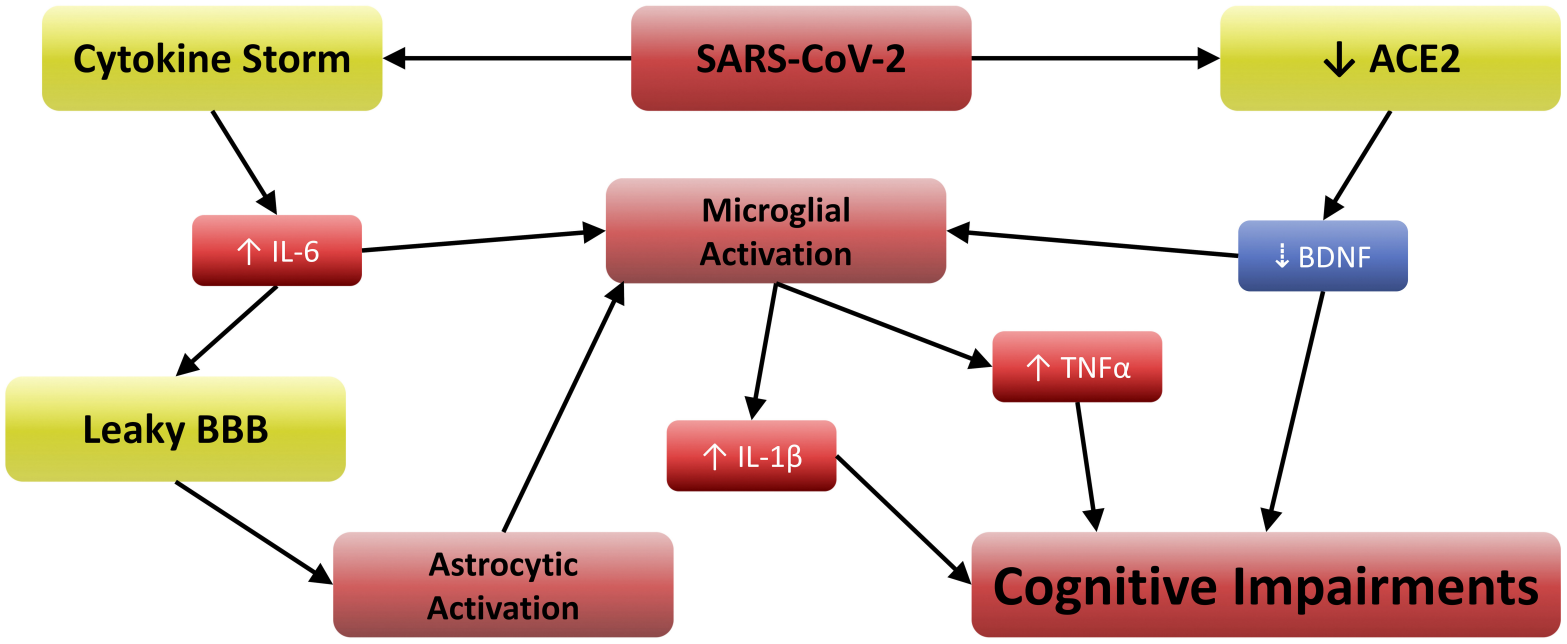
Neurobiological model for the etiology of COVID-19 related cognitive impairments.

Taken together in the context of the present study, these findings suggest that impairment of working memory and attention can both be affected by TNFα (42) and IL-1β, because both can disrupt normal firing in the neurons involved. Furthermore, these same effects would be greater in the case of clinically manifested neurotropism. In such scenarios, it is reasonable to assume that greater proportions of the microglial and astrocytic populations would be activated due to direct toll-like receptor 3/7/8 stimulation (4), and this inflammation would be furthered by reductions in BDNF (20, 36, 39). Figure 2 depicts the relationships between these variables, suggesting a well-supported neurobiological model for the etiology of COVID-19 related cognitive impairments. It is noteworthy that tests for delirium and other conditions provide categorical measures of cognitive outcomes, but a quantitative assessment such as that conducted in Zhou et al. (23) may aid future researchers in revealing the continuous cognitive effects of the neurobiological mechanism described herein. Furthermore, clinicians are urged to consider Vitamin D3 supplementation, as its active metabolite may attenuate such effects via reductions in TNFα and IL-1β production (7).

## Limitations

The main limitation of the analysis herein is a function of the limitations of the included studies (e.g., reported outcomes may have been confounded by iatrogenic effects). Sedatives are often used to treat COVID-19 patients, and other drugs may also have effects on measures of cognition. For example, research has linked chloroquine and hydroxychloroquine to psychotic symptoms and irritability (43). Other studies have linked tocilizumab to headaches, dizziness, and in some cases, strokes (44). It is also important to note that the patients included for synthesis were all hospitalized, presumably due to the severity of symptoms. Accordingly, less severe COVID-19 cases may have escaped inclusion merely due to lack of adequate reporting; a possibility which restricts the generalizability of the results reported herein. Furthermore, the diagnostic tools applied to classify cognitive impairments were nebulous in three of the included studies (as suggested in Table 4). Theoretically, this raises concerns regarding misdiagnoses which may have exaggerated the prevalence of cognitive impairments. Aside from the risk of selective reporting explained in “Implicit Reporting Bias in Prevalence Results,” this review may also be limited by the exclusivity of the search strategy. The use of the adjacency operator on EMBASE was necessary to exclude an unmanageable number of irrelevant publications, but by applying this restriction, some relevant studies may not have been identified. Nevertheless, this review provided a quantitative assessment of cognitive dysfunction associated with COVID-19 as well as a call, for both clinical and research purposes, to apply measures of cognitive function and inflammatory markers in COVID-19 patients at presentation.

## Data Availability Statement

The original contributions presented in the study are included in the article/Supplementary Materials, further inquiries can be directed to the corresponding author/s.

## Author Contributions

RM conceived of and supervised the project, among other contributions. RM and FN were responsible for managing the project and advising the team, among other contributions. YA, AS, and LL jointly determined the inclusion and exclusion criteria, screened the literature, formally assessed the quality of the included literature, and contributed to team discussions regarding data analysis. YA conducted the systematic searches, summarized the included literature, and wrote the first draft of the article. All authors contributed to team discussions regarding data analysis, synthesis, and interpretation and fact checked, edited, contributed to, and approved the submitted draft.

## Conflict of Interest

YL received salary support from the Global Alliance for Chronic Diseases/Canadian Institutes of Health Research (CIHR)/National Natural Science Foundation of China's Mental Health Team Grant and the CIHR Frederick Banting and Charles Best Canada Graduate Scholarship; personal fees from Champignon Brands. RM has received research grant support from CIHR/GACD/Chinese National Natural Research Foundation; speaker/consultation fees from Lundbeck, Janssen, Purdue, Pfizer, Otsuka, Allergan, Takeda, Neurocrine, Sunovion, Minerva, Intra-Cellular, Abbvie, and Eisai. RM is a shareholder and CEO of Champignon. JR has received research grant support from the Canadian Cancer Society, Canadian Psychiatric Association, American Psychiatric Association, American Society of Psychopharmacology, University of Toronto, University Health Network Centre for Mental Health, Joseph M. West Family Memorial Fund and Timeposters Fellowship and industry funding for speaker/consultation/research fees from Allergan, Lundbeck and COMPASS. JR is the medical director of a private clinic providing off-label ketamine infusions for depression. The remaining authors declare that the research was conducted in the absence of any commercial or financial relationships that could be construed as a potential conflict of interest.
